# Clinical Profile and Factors Associated With Acute Kidney Injury in Critically Ill Patients Admitted to a Tertiary Care Intensive Care Unit in Bangladesh

**DOI:** 10.7759/cureus.109301

**Published:** 2026-05-20

**Authors:** Md. Kabir Hossain, Syed Fazlul Islam, Rana Mokaram Hossain, Ferdous Jahan, Md. Abdul Muqueet, Md. Kamal Uddin, S. M. Remin Rafi, Md. Ahsan Ullah, Md. Masudul Karim

**Affiliations:** 1 Department of Nephrology, Bangladesh Medical University (BMU), Dhaka, BGD; 2 Department of Nephrology, Ibn Sina Medical College Hospital, Dhaka, BGD; 3 Department of Critical Care Medicine, Dhaka Medical College Hospital, Dhaka, BGD; 4 Department of Critical Care Medicine (ICU), Islami Bank Central Hospital, Dhaka, BGD

**Keywords:** acute kidney injury, bangladesh, intensive care unit, kdigo criteria, prospective study, septic shock

## Abstract

Background

Acute kidney injury (AKI) is a life-threatening complication among critically ill patients, particularly in resource-limited settings such as Bangladesh, where standardized diagnostic protocols and ICU infrastructure remain inadequate. Evidence on its clinical profile and associated factors in tertiary care ICUs in Bangladesh is scarce. The primary objective of this study was to describe the clinical profile and burden of AKI among critically ill adult patients admitted to a tertiary care ICU in Bangladesh. The secondary objective was to explore clinical and laboratory factors associated with AKI.

Methods

A prospective observational study was conducted at the ICU of Islami Bank Central Hospital, Dhaka, Bangladesh, from January to December 2024. A total of 70 critically ill adult patients (aged ≥18 years) were enrolled via consecutive sampling. AKI was diagnosed via the Kidney Disease: Improving Global Outcomes (KDIGO) criteria. Data on sociodemographic characteristics, comorbidities, hemodynamic and laboratory parameters, and clinical interventions were collected via structured data collection sheets. Univariate and multivariate logistic regression analyses were performed via STATA to identify potential factors associated with AKI. A p-value of less than 0.05 was considered statistically significant.

Results

The overall incidence of AKI was 42 (60.0%). The mean age of the participants was 62.17 ± 17.88 years; 38 (54.3%) were male. Common comorbidities included hypertension in 40 patients (57.1%), diabetes mellitus in 37 patients (52.9%), and chronic kidney disease in 38 patients (54.3%). Sepsis was present in 48 patients (68.6%), and 26 patients (37.1%) developed septic shock. According to multivariate analysis, higher hemoglobin was associated with lower odds of AKI (AOR = 0.49; 95% CI: 0.28-0.85; p = 0.011), whereas hypertension (AOR = 14.02; 95% CI: 2.74-71.87; p = 0.002), septic shock (AOR = 7.68; 95% CI: 1.43-41.21; p = 0.017), and chronic health conditions (AOR = 9.45; 95% CI: 1.86-47.89; p = 0.007) were associated with higher odds of AKI.

Conclusions

A high burden of AKI was observed among critically ill ICU patients in Bangladesh. Higher hemoglobin levels were associated with lower odds of AKI, whereas hypertension, septic shock and chronic health conditions were associated with higher odds in the exploratory adjusted analysis. Given the limited sample size and exploratory nature of the analysis, these findings should be interpreted cautiously and validated through larger multicenter studies incorporating AKI staging and outcome assessment.

## Introduction

Acute kidney injury (AKI) is a major global clinical concern because of its substantial effect on patient outcomes and healthcare systems. It is characterized by an abrupt decline in kidney function, resulting in metabolic waste accumulation and fluid imbalance. Among hospitalized patients, AKI is associated with increased morbidity, mortality, and subsequent progression to chronic kidney disease (CKD) [1]. Its burden is particularly high in critically ill patients. A large multinational study reported that AKI occurs in more than half of ICU patients, with a pooled incidence of 57.3% among critically ill adults [2]. Severe AKI, especially when renal replacement therapy (RRT) is required, is associated with high mortality, prolonged ICU and hospital stay, and increased healthcare costs [3].

In the ICU, AKI commonly develops as part of an underlying critical illness. Sepsis is one of the leading contributors to ICU-acquired AKI and is associated with worse outcomes than nonseptic AKI [4]. Hemodynamic instability, systemic inflammation, nephrotoxic exposure, cardiogenic shock, hypovolemia, and vasoactive drug use may all impair renal perfusion and contribute to ischemic or inflammatory renal injury [5]. Persistent hypotension, particularly when mean arterial pressure falls below 65 mmHg, can reduce renal blood flow and promote kidney injury [6]. Sepsis-related endothelial dysfunction, microvascular thrombosis, and tubular injury further contribute to the development of AKI [7]. In addition, preexisting conditions such as hypertension, diabetes mellitus, and CKD may reduce renal reserve and increase vulnerability to acute renal insult [7].

Several clinical and laboratory factors observed during ICU admission may also be associated with AKI. Reduced hemoglobin may impair oxygen delivery to renal tissue and contribute to renal ischemia [8]. The need for invasive mechanical ventilation and vasopressor support often reflects greater illness severity and circulatory instability. Previous studies have identified vasopressor use as an important factor associated with AKI development [9]. Septic shock, compared with sepsis without shock, has also been associated with markedly higher odds of AKI [10].

The burden of AKI is disproportionately high in low- and middle-income countries, where ICU beds, RRT facilities, and diagnostic resources are often limited [11]. In South Asia, both community-acquired and hospital-acquired AKI remain important clinical problems and are often associated with substantial mortality [12]. In Bangladesh, ICU resources are limited, and standardized AKI prevention and management protocols are not uniformly implemented. Available local evidence suggests a considerable burden of AKI among critically ill patients. A tertiary care ICU study in Dhaka reported that 21.77% of admitted patients developed AKI [13], while studies from critical care nephrology settings in Bangladesh have reported even higher frequencies in selected populations [14]. However, evidence using standardized contemporary diagnostic criteria among adult ICU patients in Bangladesh remains limited.

Early recognition of AKI and its associated clinical factors is important for timely monitoring, optimization of hemodynamic status, avoidance of nephrotoxic drugs, and correction of fluid and electrolyte imbalance [15]. Locally generated evidence is particularly important because patient profiles, comorbidity patterns, timing of hospital presentation, and ICU resources may differ from those in high-income settings. Therefore, the primary objective of this study was to describe the clinical profile and burden of AKI among critically ill adult patients admitted to a tertiary care ICU in Bangladesh. The secondary objective was to explore clinical and laboratory factors associated with AKI in this population.

## Materials and methods

Study design and place

A prospective observational study was conducted in the intensive care unit (ICU) of Islami Bank Central Hospital, Kakrail, Dhaka, Bangladesh, from January to December 2024.

Study population and eligibility criteria

Critically ill adult patients admitted to the ICU during the study period were considered for enrollment. Critically ill patients were defined as patients requiring ICU admission for close monitoring or organ support because of life-threatening illness, hemodynamic instability, respiratory failure, sepsis, or other acute clinical deterioration, as determined by the treating ICU team. Patients aged 18 years or older, irrespective of sex, were eligible for inclusion if they had sufficient clinical and laboratory information to allow assessment of renal function. Patients with preexisting end-stage renal disease on maintenance dialysis, a history of renal transplantation, age below 18 years, or incomplete essential clinical or laboratory records required for AKI classification were excluded. During the study period, 85 critically ill adult patients were screened for eligibility. Of these, 15 patients were excluded because of end-stage renal disease on maintenance dialysis, history of renal transplantation, or incomplete essential records. Finally, 70 patients were included in the analysis using a consecutive sampling technique.

Sample size and sampling technique

The minimum required sample size was calculated via the following formula:

\[ n=\frac{Z^2pq}{d^2} \]

Based on previous data from Bangladesh, the prevalence of AKI was assumed to be 21.77% [13]. Using a 95% confidence level (Z = 1.96) and a margin of error of 10%, the calculated minimum sample size was 66. To enhance representation and compensate for possible exclusions due to incomplete data, a total of 70 patients were finally included. Participants were selected using a consecutive sampling technique.

Measures and definitions

AKI was diagnosed according to the Kidney Disease: Improving Global Outcomes (KDIGO) criteria, defined as an increase in serum creatinine of ≥0.3 mg/dL within 48 hours, an increase in serum creatinine to ≥1.5 times the baseline value within the preceding seven days, or urine output of <0.5 mL/kg/hour for at least six hours [16]. AKI was assessed at ICU admission and during subsequent ICU follow-up using available serum creatinine and urine output data. Because the exact timing of AKI onset before ICU admission could not be determined in all cases, AKI was not separately analyzed as community-acquired or ICU-acquired.

Baseline serum creatinine was obtained from available preadmission medical records whenever present. For patients without documented preadmission creatinine values, admission serum creatinine was used cautiously as the reference value. Serial serum creatinine values during ICU stay were reviewed for AKI classification according to KDIGO criteria. Preexisting CKD was identified from documented medical history, previous laboratory evidence of chronic renal impairment, or physician diagnosis recorded in the medical files. CKD staging could not be performed uniformly because previous estimated glomerular filtration rate or serial creatinine values were not available for all patients.

Urine output was recorded from ICU nursing charts. In catheterized patients, hourly urine output was documented and summarized over six-hour and 24-hour periods. In non-catheterized patients, urine output was recorded from measured urine collection when available. Oliguria was defined as reduced urine output of <400 mL over 24 hours [17]. This term was used to indicate reduced urine output and did not refer to anuria, which denotes absent or near-absent urine output.

Preexisting comorbidities, including hypertension, diabetes mellitus, ischemic heart disease, and CKD, were identified from documented medical records. A chronic health condition was considered present when a patient had two or more documented preexisting comorbidities among hypertension, diabetes mellitus, ischemic heart disease, and CKD. Sepsis was defined as life-threatening organ dysfunction resulting from a dysregulated host response to infection, indicated by an increase of ≥2 points in the Sequential Organ Failure Assessment (SOFA) score [18]. Septic shock was defined as sepsis with persistent hypotension requiring vasopressor therapy to maintain a mean arterial pressure (MAP) of ≥65 mmHg, along with a serum lactate level >2 mmol/L despite adequate fluid resuscitation [18]. Hemodynamic variables, including MAP, heart rate, and respiratory rate, were recorded at ICU admission. The requirement for mechanical ventilation and vasoactive medications was also documented as markers of illness severity.

Data collection procedure

Data were collected using a predesigned data collection form through direct patient evaluation and review of hospital records. Written informed consent was obtained from patients or their legal guardians before data collection. Demographic information, relevant clinical history, preexisting comorbidities, and ICU-related clinical parameters were documented. The observed clinical variables included age, sex, Glasgow Coma Scale (GCS) score, MAP, heart rate, respiratory rate, urine output, presence of sepsis or septic shock, requirement for mechanical ventilation, use of vasoactive medications, and length of ICU stay. Laboratory variables included admission values of complete blood count, hemoglobin, hematocrit, serum creatinine, serum sodium, serum potassium, arterial pH, and bicarbonate level. Hemoglobin and other baseline laboratory parameters used in comparative and regression analyses represented admission values unless otherwise specified. For serum creatinine, both admission and subsequent values during ICU stay were reviewed for AKI classification. All participants were followed throughout their ICU stay to observe the occurrence of AKI.

Statistical analysis

All collected data were reviewed for completeness before analysis and processed using STATA software version 15.0 (StataCorp LLC, College Station, Texas, USA). Patients with incomplete essential clinical or laboratory data required for AKI classification were excluded. No imputation was performed for missing data. Continuous variables were summarized as mean ± standard deviation, while categorical variables were presented as frequency and percentage. The normality of continuous variables was assessed using the Shapiro-Wilk test. Comparisons between patients with and without AKI were performed using the independent samples t-test for normally distributed continuous variables and the Mann-Whitney U test for non-normally distributed variables. Categorical variables were compared using the chi-square test or Fisher’s exact test, as appropriate. To explore factors associated with AKI, univariate logistic regression analysis was first performed. Variables with p<0.05 in univariate analysis, together with selected clinically relevant variables based on prior evidence, were considered for multivariable logistic regression. Considering the limited number of patients in the smaller outcome group, the multivariable model was kept parsimonious to reduce the possibility of overfitting. Clinical overlap among candidate variables was also considered, and highly overlapping variables were not entered together where possible. The final exploratory multivariable model included hemoglobin, hypertension, septic shock, and chronic health conditions. Adjusted odds ratios (AORs) with corresponding 95% confidence intervals (CIs) were reported. The findings from the multivariable model were interpreted as exploratory associations rather than definitive predictors. A p-value of <0.05 was considered statistically significant.

Ethical considerations

Ethical clearance for this study was granted by the Institutional Ethical Review Board of the Centre for Medical Research and Development (Approval no: CMRD/IRB/2023/137). The study procedures were carried out in line with the ethical standards outlined in the Declaration of Helsinki. Before participation, written informed consent was obtained from all patients or their authorized representatives. Throughout the study period, strict measures were taken to ensure the confidentiality and anonymity of all collected patient information.

## Results

Baseline characteristics of the participants

The mean age of the participants was 62.17 years (SD ± 17.88), with the majority aged more than 55 years (50; 71.4%). Male patients constituted 38 (54.3%) of the study population, whereas 32 (45.7%) were female. AKI was observed in 42 (60.0%) of patients. A substantial proportion of patients had preexisting comorbidities, including hypertension 40 (57.1%), diabetes mellitus 37 (52.9%), ischemic heart disease 36 (51.4%), and CKD 38 (54.3%). Sepsis was present in 48 (68.6%) patients, and 26 (37.1%) developed septic shock. More than half of the patients required mechanical ventilation 39 (55.7%) or vasoactive drugs 46 (65.7%). Oliguria was observed in 47 (67.1%) of the patients, while 34 (48.6%) had underlying chronic health conditions. A high proportion (59; 84.3%) had a serum creatinine concentration ≥1.2 mg/dL at presentation (Table 1).

**Table 1:** Baseline characteristics of the study participants (n=70)

Variables	n (%)
Male	38 (54.3)
Female	32 (45.7)
Age >55 years	50 (71.4)
Hypertension	40 (57.1)
Diabetes mellitus	37 (52.9)
Ischemic heart disease	36 (51.4)
Chronic kidney disease	38 (54.3)
Sepsis	48 (68.6)
Septic shock	26 (37.1)
Mechanical ventilation	39 (55.7)
Vasoactive drugs	46 (65.7)
Oliguria (<400 mL/24 h)	47 (67.1)
Chronic health condition	34 (48.6)
Serum creatinine ≥1.2 mg/dL at admission	59 (84.3)
Acute kidney injury	42 (60.0)

Clinical and laboratory profiles of the participants

The clinical and laboratory parameters of the study participants are summarized in Table 2. The mean age was 62.17 ± 17.88 years. The mean GCS score was 12.20 ± 3.56, suggesting moderate impairment of consciousness. The mean hemoglobin level was 7.00 ± 1.74 g/dL, indicating that the study population was predominantly anemic. The mean serum creatinine level was 2.81 ± 1.71 mg/dL. The average duration of ICU stay was 7.20 ± 5.48 days. The mean MAP was 75.90 ± 20.13 mmHg, and the mean heart rate was 102.17 ± 32.08 beats per minute. The mean respiratory rate was 23.16 ± 8.40 breaths per minute. The electrolyte levels were relatively within normal limits, with a mean sodium level of 136.63 ± 7.57 mmol/L and a mean potassium level of 4.10 ± 0.98 mmol/L. The mean arterial pH was 7.24 ± 0.06, and the bicarbonate level was 18.03 ± 3.39 mmol/L, indicating a tendency toward metabolic acidosis. The mean hematocrit was 21.0 ± 5.2%.

**Table 2:** Clinical and laboratory profiles of the study participants (n=70) GCS: Glasgow Coma Scale; MAP: mean arterial pressure

Variables	Mean ± SD
Age (years)	62.17 ± 17.88
GCS score	12.20 ± 3.56
Hemoglobin (g/dL)	7.00 ± 1.74
Creatinine (mg/dL)	2.81 ± 1.71
ICU stay (days)	7.20 ± 5.48
MAP (mmHg)	75.90 ± 20.13
Heart rate (beats/min)	102.17 ± 32.08
Respiratory rate (breaths/min)	23.16 ± 8.40
Sodium (mmol/L)	136.63 ± 7.57
Potassium (mmol/L)	4.10 ± 0.98
pH	7.24 ± 0.06
HCO3 (mmol/L)	18.03 ± 3.39
Hematocrit (%)	21.0 ± 5.2

Comparison between the AKI and non-AKI groups

Table 3 shows the comparison of clinical parameters between patients with and without AKI. Hemoglobin levels were significantly lower in the AKI group than in the non-AKI group (6.39 ± 1.19 vs 7.92 ± 2.05, p<0.001). Patients with AKI had significantly lower MAPs (71.02 ± 21.03 vs 83.21 ± 16.47, p=0.012) and higher heart rates (109.71 ± 33.15 vs 90.86 ± 27.19, p=0.015). The respiratory rate was also significantly greater in the AKI group (24.79 ± 8.68 vs 20.71 ± 7.45, p=0.046). No significant differences were observed in age, admission serum creatinine level or ICU stay between the two groups.

**Table 3:** Comparison of characteristics between the AKI and non-AKI groups MAP: mean arterial pressure; ICU: intensive care unit; AKI: acute kidney injury. Serum creatinine represents the value measured at ICU admission; *p < 0.05 considered statistically significant

Variables	No AKI (n=28)	AKI (n=42)	p value
Age (years)	60.61 ± 21.03	63.21 ± 15.62	0.554
Hemoglobin (g/dL)	7.92 ± 2.05	6.39 ± 1.19	<0.001*
Serum creatinine at admission (mg/dL)	2.77 ± 1.76	2.84 ± 1.69	0.859
ICU stay (days)	5.86 ± 4.23	8.10 ± 6.06	0.094
MAP (mmHg)	83.21 ± 16.47	71.02 ± 21.03	0.012*
Heart rate (beats/min)	90.86 ± 27.19	109.71 ± 33.15	0.015*
Respiratory rate (breaths/min)	20.71 ± 7.45	24.79 ± 8.68	0.046*

Factors associated with AKI

The univariate logistic regression analysis results are presented in Table 4. Higher hemoglobin levels were significantly associated with lower odds of AKI (OR=0.55, 95% CI: 0.38-0.79; p=0.001). Hemodynamic instability markers, such as lower MAP (OR=0.97, p=0.015), higher heart rate (OR=1.02, p=0.018) and higher respiratory rate (OR=1.06, p=0.049), were also significantly associated with AKI. Among the comorbidities, hypertension (OR=11.00, p<0.001), diabetes mellitus (OR=4.22, p=0.006), chronic kidney disease (OR=3.60, p=0.012), septic shock (OR=6.60, p=0.002) and chronic health conditions (OR=7.33, p<0.001) were significantly associated with AKI development.

**Table 4:** Univariate logistic regression analysis for factors associated with AKI MAP: Mean arterial pressure; AKI: acute kidney injury *p < 0.05 considered statistically significant

Variables	OR	95% CI	p-value
Hemoglobin	0.55	0.38–0.79	0.001*
MAP	0.97	0.94–0.99	0.015*
Heart rate	1.02	1.00–1.04	0.018*
Respiratory rate	1.06	1.00–1.13	0.049*
Hypertension	11.00	3.56–34.02	<0.001*
Diabetes mellitus	4.22	1.52–11.71	0.006*
Chronic kidney disease	3.60	1.32–9.83	0.012*
Septic shock	6.60	1.95–22.34	0.002*
Chronic health condition	7.33	2.42–22.20	<0.001*

Exploratory multivariable analysis of factors associated with AKI

The results of the exploratory multivariable logistic regression analysis are presented in Table 5. After adjustment, higher hemoglobin level remained significantly associated with lower odds of AKI (AOR=0.49, 95% CI: 0.28-0.85, p=0.011). In contrast, hypertension was significantly associated with increased odds of AKI (AOR=14.02, 95% CI: 2.74-71.87, p=0.002). Septic shock also showed a significant positive association with AKI (AOR=7.68, 95% CI: 1.43-41.21, p=0.017). Additionally, the presence of chronic health conditions was associated with greater odds of AKI (AOR=9.45, 95% CI: 1.86-47.89, p=0.007).

**Table 5:** Exploratory multivariable logistic regression analysis for factors associated with AKI *p < 0.05 considered statistically significant; AKI: acute kidney injury

Variables	Adjusted OR	95% CI	p-value
Hemoglobin	0.49	0.28–0.85	0.011*
Hypertension	14.02	2.74–71.87	0.002*
Septic shock	7.68	1.43–41.21	0.017*
Chronic health condition	9.45	1.86–47.89	0.007*

## Discussion

The present study described the clinical profile and burden of AKI and explored factors associated with AKI among critically ill ICU patients in Bangladesh. AKI was observed in 60.0% of patients. Higher hemoglobin level was associated with lower odds of AKI, while hypertension, septic shock and chronic health conditions were associated with higher odds in the exploratory adjusted analysis. Other clinical parameters did not remain significant after adjustment.

The 60.0% incidence of AKI observed in this study is consistent with the high prevalence reported in critically ill populations globally. A large multinational study involving over 1802 ICU patients reported an AKI incidence of 57.3% according to the KDIGO criteria, with sepsis and hemodynamic instability as predominant contributors [2]. Similarly, a systematic review of AKI in South Asian ICUs reported pooled incidences ranging from 45% to 65%, with higher rates observed in settings where baseline renal impairment and delayed presentation are common [19]. Specifically, in Bangladesh, a prospective study from a tertiary care hospital reported that 49.7% of critically ill patients developed AKI, a figure closely aligned with the present findings [20]. The high burden of AKI in this cohort likely reflects the confluence of late hospital admission, limited pre-ICU resuscitative resources, and a high prevalence of underlying comorbidities such as hypertension and diabetes, which compromise the renal reserve and render the kidney more susceptible to acute insults [19].

One notable finding was the association between higher hemoglobin levels and lower odds of AKI in the exploratory adjusted analysis. This inverse relationship is biologically plausible. Anemia reduces the arterial oxygen content, compromising oxygen delivery to the highly metabolically active renal medulla, which operates under physiologically hypoxic conditions [21]. This finding is consistent with prior studies demonstrating that lower hemoglobin levels increase the odds of AKI, thereby supporting the protective association of higher hemoglobin observed in our study [22]. A hemoglobin threshold below 7 g/dL has been independently associated with AKI development [22]. The mean hemoglobin level of 7.00 g/dL in the present study was notably low, and the significantly lower hemoglobin level in the AKI group (6.39 vs 7.92 g/dL) suggested that even modest differences in the oxygen-carrying capacity may influence renal outcomes. While some studies have reported weaker or inconsistent associations after adjustment for illness severity, these findings suggest that hemoglobin may be a clinically relevant marker, although this association requires cautious interpretation [23].

Preexisting hypertension was also associated with higher odds of AKI in the exploratory adjusted model. A meta-analysis of 45 cohort studies revealed that preexisting hypertension increased the odds of AKI by approximately 38% (pooled OR 1.38, 95% CI 1.21-1.57) in hospitalized patients [24]. The substantially high odds ratio observed in the present study may reflect the additive effect of hypertension with other unmeasured factors, such as long-term use of renin‒angiotensin system blockers, poorer baseline renal function, or a greater burden of microvascular disease in this specific population.

Septic shock was associated with higher odds of AKI in the exploratory adjusted analysis, supporting the role of sepsis-related hemodynamic and inflammatory injury in AKI. The comparison between the AKI and non-AKI groups in this study revealed significantly lower MAPs and higher heart rates among patients who developed AKI, which is consistent with the clinical picture of distributive shock. Septic shock contributes to AKI through multiple synergistic pathways: systemic vasodilation leading to renal hypoperfusion, activation of inflammatory cascades that cause endothelial dysfunction and microcirculatory thrombosis, and direct tubular injury from pathogen-associated molecular patterns [25]. A large international cohort study revealed that septic shock patients had significantly higher AKI rates than nonshock sepsis patients did [26]. The even stronger association observed in our study may reflect the particularly high burden of delayed sepsis recognition and limited access to early goal-directed therapy in resource-constrained settings. The finding that the respiratory rate was significantly greater in the AKI group further supports the notion that AKI develops in the context of greater overall physiological derangement rather than as an isolated renal event.

The presence of chronic health conditions was associated with higher odds of AKI, reflecting a reduced physiological reserve and increased vulnerability. Evidence from a large MIMIC-IV cohort revealed that higher Charlson Comorbidity Index scores independently predict mortality (HR 1.277, 95% CI 1.233-1.323), highlighting the impact of baseline health status [27]. This association may reflect the contribution of cumulative comorbidity burden; however, overlap between the composite chronic health condition variable and individual comorbidities may have influenced the adjusted estimate. This finding aligns with the concept of frailty and reduced renal reserve, where the aging or chronically diseased kidney has a diminished capacity to withstand acute insults such as hypotension, sepsis, or nephrotoxin exposure [28].

The strengths of this study include its prospective observational design, real-world ICU setting, use of KDIGO criteria, and inclusion of demographic, clinical, laboratory, and ICU-related variables to describe the burden and clinical profile of AKI in a resource-limited Bangladeshi setting. However, several limitations should be noted. This was a single-center study with a small sample size, which may limit generalizability. Although the sample size was appropriate for describing AKI burden, the study was not primarily powered for multivariable regression; therefore, the adjusted estimates may be imprecise, as reflected by wide confidence intervals, and should be interpreted as exploratory associations. Baseline renal function was not uniformly available, so use of admission creatinine may have caused misclassification of AKI or difficulty distinguishing AKI, AKI on CKD, and previously unrecognized CKD. CKD staging, AKI staging, renal recovery, mortality, and long-term outcomes were not assessed. Formal ICU severity scores were also unavailable, and causal relationships cannot be inferred. Larger multicenter studies with standardized baseline renal assessment, AKI staging, illness severity scoring, and outcome follow-up are needed to validate these findings.

## Conclusions

The present study demonstrated a high burden of AKI among critically ill ICU patients in Bangladesh. Higher hemoglobin levels were associated with lower odds of AKI, whereas preexisting hypertension, septic shock and greater comorbidity burden showed significant associations with higher odds of AKI in the exploratory adjusted analysis. These findings emphasize the importance of early identification of clinically vulnerable patients and careful monitoring of renal function in the ICU. Strengthening preventive strategies and optimizing hemodynamic and supportive care may help reduce the occurrence and adverse outcomes of AKI in resource-limited critical care settings.
